# Supplementary material for: Amino acid dependent formaldehyde metabolism in mammals
Source: Commun Chem. 2020 Jun 16;3:78. doi: 10.1038/s42004-020-0324-z (PMC9814826; doi:10.1038/s42004-020-0324-z)
Supplement: Supplementary file 1 — Supplementary Information [file 42004_2020_324_MOESM1_ESM.pdf]

Amino acid dependent formaldehyde metabolism in mammals

Supplementary Information

Matthias Pietzke[1], Guillermo Burgos-Barragan[2], Niek Wit[2], Jacqueline Tait-Mulder[1], David Sumpton[1], Gillian M Mackay[1], Ketan J. Patel[2], Alexei Vazquez[1,2*]

[1]Cancer Research UK Beatson Institute, Switchback Road, Bearsden, Glasgow, G61 1BD UK

[2]MRC Laboratory of Molecular Biology, Francis Crick Avenue, Cambridge, CB2 0QH, UK

[3]Institute of Cancer Sciences, University of Glasgow, Switchback Road, Bearsden, Glasgow, G61 1QH, UK

*Correspondence to:

Alexei Vazquez

Cancer Research UK Beatson Institute

Switchback Road, Bearsden, Glasgow, G61 1BD, UK

Email: alexei.vazquezvazquez@glasgow.ac.uk

This PDF file includes:

Formaldehyde quantification

Supplementary Figure 1

Supplementary Tables 1 and 2

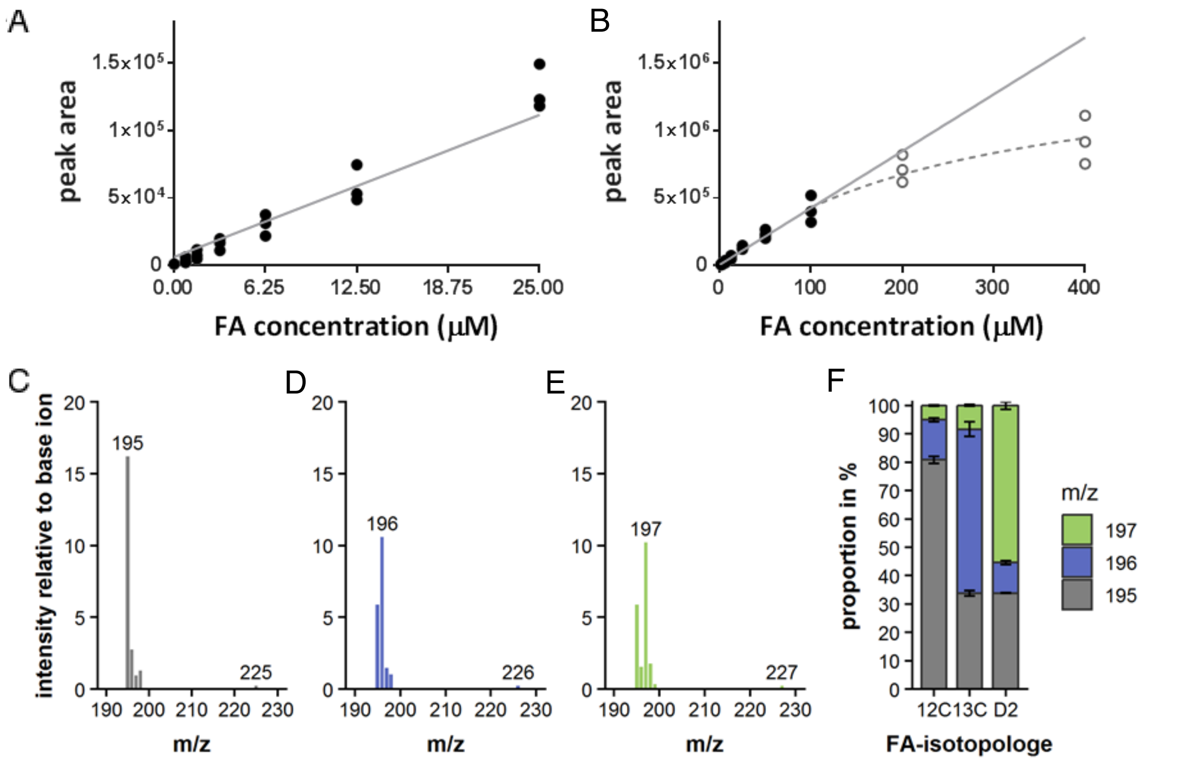

Supplementary Figure 1

A,B) Low (A) and high (B) concentration range of figure 1C, emphasizing the linearity up to 100 μM formaldehyde. C-F) Isotopic interference between the formaldehyde-PFBHA fragments containing the formaldehyde backbone in the m/z range from 190 to 230: m/z of ^{12}C-formaldehyde (C), ^{13}C-formaldehyde (D) and D2-formaldehyde (E). F) Proportion of each of the fragments within the range of 195 to 197. Evident is a constant fraction of ^{12}C (m/z = 195) in all cases, indicating an additional fragment that doesn't contain the formaldehyde carbon.

m/z 195 (Peak area)	m/z 196 (Peak area)	mz/197 (Peak area)	F_0 (mM)	F_1 (mM)	F_2 (mM)
50022.0112	11486.75889	84305.68527	0	0	0.01
67369.21804	13476.80554	79063.73855	0.001	0	0.01
88591.57913	14845.47327	80406.57654	0.003	0	0.01
118436.0286	18691.82262	76580.53826	0.006	0	0.01
178219.3993	26251.11177	77465.08001	0.012	0	0.01
344824.8688	45413.88982	94706.31722	0.025	0	0.01
610068.1277	72793.02112	104234.4871	0.05	0	0.01
1166748.891	138147.9644	125521.4035	0.1	0	0.01
57097.82014	22599.72822	81744.69495	0	0.001	0.01
61827.10108	35192.92003	82000.37224	0	0.003	0.01
73427.09768	60033.15332	88396.43395	0	0.006	0.01
95533.70968	110621.1491	95946.38733	0	0.012	0.01
159262.0484	229627.5876	121690.6415	0	0.025	0.01
252020.2936	429709.2193	132336.6509	0	0.05	0.01
509136.1414	955266.9226	209248.586	0	0.1	0.01
54239.56108	11096.08687	89094.8986	0	0	0.01
80067.76302	14982.0862	95708.224	0.001	0	0.01
86940.89952	15693.203	85870.54207	0.003	0	0.01
133712.8038	19758.57228	90335.71983	0.006	0	0.01
192915.7721	26685.84261	89471.26188	0.012	0	0.01
360336.3492	47477.16199	106263.0843	0.025	0	0.01
710946.5161	86073.05134	123796.8851	0.05	0	0.01
1242834.645	140025.7089	138803.6856	0.1	0	0.01
59243.73696	25716.50016	93119.38244	0	0.001	0.01
64634.37403	39993.51315	94488.53158	0	0.003	0.01
80821.45506	64618.79223	103494.651	0	0.006	0.01
103314.1851	119191.9921	103329.0836	0	0.012	0.01
147883.1333	217449.5561	109493.5828	0	0.025	0.01
265667.4009	448600.2819	145191.608	0	0.05	0.01
500684.9384	933911.3867	214067.7842	0	0.1	0.01
52137.68543	11390.75128	89863.84098	0	0	0.01
73312.09223	14818.61165	90201.17776	0.001	0	0.01
98866.15264	16801.5797	95371.96103	0.003	0	0.01
132255.1796	21283.84233	94091.42805	0.006	0	0.01
218905.7898	30477.11722	100843.3232	0.012	0	0.01
344588.1321	44912.28766	101251.8323	0.025	0	0.01
643304.7357	77833.66983	112937.9941	0.05	0	0.01
1242574.765	137117.4354	137546.864	0.1	0	0.01
57365.81475	23314.0875	85178.03193	0	0.001	0.01
68419.47312	38400.65767	99142.89642	0	0.003	0.01
78457.25626	63535.70559	98130.51581	0	0.006	0.01
92997.68031	107079.2725	98253.55779	0	0.012	0.01
153709.1975	222578.709	107895.1161	0	0.025	0.01
257049.6529	423045.7406	149233.0386	0	0.05	0.01
438415.5227	824217.5172	191300.0782	0	0.1	0.01

Supplementary Table 1. Data used to train the formaldehyde quantification model.

Compound	IUPAC name	PubChem -ID	Sum formula	measured m/z	polarity	retent ion time	validated with authentic standard?	M+1 when 13C was supplied?
Timonacic	thiazolidine-4-carboxylic acid	9934	$C_4H_7NO_2S$	132.0125 (M-H), 134.0271 (M+H)	neg / pos	2.6	yes	yes
N-formyl-Cysteine	N-formyl-L-cysteine	29649	$C_4H_7NO_3S$	148.0074 (M-H) , 149.0109 (M+H)	neg / pos	2.6		yes
Spinacine	(6*S*)-4,5,6,7-tetrahydro-3*H*-imidazo[4,5-c]pyridine-6-carboxylic acid	162899	$C_7H_9N_3O_2$	168.0767 (M+H)	neg / pos	7.3		yes
N-methyl-Glutamate	N-methyl-L-glutamic acid	439377	$C_6H_{11}NO_4$	…	…	…		…

Supplementary Table 2. Parameters for the identification of previous unknown compounds by LC-MS.
